# Mediating Effects of Exposure to Violence in Different Contexts of Child-to-Parent Violence: Validation of the Exposure to Violence Scale

**DOI:** 10.3390/children12040409

**Published:** 2025-03-24

**Authors:** Luis Burgos-Benavides, M. Carmen Cano-Lozano, Isabel Suevos-Rodríguez, Paola Bustos-Benítez, Francisco Javier Rodríguez-Díaz

**Affiliations:** 1 Department of Psychology, University of Oviedo, 33003 Oviedo, Spain; isabelsuevos@correo.ugr.es (I.S.-R.); gallego@uniovi.es (F.J.R.-D.); 2 Department of Psychology, University of Jaen, 23071 Jaen, Spain; mccano@ujaen.es; 3 Department of Psychology, Fundación Universitaria Konrad Lorenz, Bogotá 110231, Colombia; paolar.bustosb@konradlorenz.edu.co

**Keywords:** exposure to violence, child-to-parent violence, reasons, psychometric properties, measurement invariance, adolescent

## Abstract

Exposure to violence is one of the most common adverse effects. In recent years, there has been a particular interest in understanding the link between exposure to violence and the perpetration of other forms of violence, such as child-to-parent violence, a complex family problem that severely affects the relationships between family members. Objective: We aimed to examine the mediating role of instrumental and reactive reasons in the relationship between exposure to violence and child-to-parent violence in different contexts. To fulfill this objective, it was necessary to analyze the evidence of validity and psychometric properties of the Violence Exposure Scale with Colombian adolescents. Methods: A total of 981 Colombian adolescents between aged 12 and 18 years participated. The participants responded to two psychometric scales: one on exposure to violence and the other on child-to-parent violence. Results: The Violence Exposure Scale presented an excellent psychometric model and evidence of adequate validity and reliability. Girls reported greater exposure to violence at home and boys reported greater exposure to violence at school and on the street. In general, older adolescents reported greater exposure to domestic violence. Instrumental and reactive reasons were significant predictors in the perpetuation of child-to-parent violence, with victimization in the home being the best predictor and reactive reasons the strongest mediating variables. Conclusions: Exposure to violence in the home is a key predictor; however, the co-occurrence of violence in other settings plays an important role in polyvictimization and predicting future violent behavior.

## 1. Introduction

Studies on exposure to violence have advanced over the last seven years [1]. It is estimated that approximately 300 million children (under the age of 18) are exposed to violence worldwide. Exposure to violence before the age of 18 years is a type of adverse childhood experience in which the child experiences a stimulus of violence in his or her life [2]. Exposure to violence can take place in different scenarios, and it is common for it to interact with other adverse experiences such as abuse, neglect, or substance use [3,4,5]. In addition, adverse experiences can occur in a variety of contexts, such as the home, school, community, or neighborhood [3,6,7,8,9], and in the last few years, through technological means [2,10,11]. Additionally, victimization may overlap by co-occurring in parallel in different settings, thus increasing the risk of polyvictimization [9,12,13].

According to some authors [14,15,16,17,18], exposure to violence occurs in two ways: direct victimization and vicarious victimization. In direct victimization, children are directly victimized through physical, emotional, and sexual abuse or neglect. In vicarious victimization, children observe violent behaviors toward others who may be family or non-family members. The findings show that the impact of exposure to violence varies according to the type of victimization and scenario [19]. However, exposure to intimate partner violence is considered direct emotional abuse, because children who witness violence against the mother may be as affected as if they were experiencing direct victimization [16].

Also, exposure to violence at home is the earliest adverse experience and appears to be the most harmful, as it puts children’s safety and health at risk [6], mainly because this experience occurs in the child’s immediate environment and has the effect of distorting the child’s sense of safety and security. Therefore, it is likely to generate a sense of insecurity and loss of stability and well-being, which mainly affects positive and stable relationships [17]. For example, children are more affected when they witness violence toward a family member. However, individuals who have experienced recurrent exposure to violence are more likely to engage in future violent behavior [20].

Exposure to violence is commonly considered one of the most controversial predictors, as it has been shown to have broadly complex effects on mental health, psychopathology, and links to the perpetuation of subsequent criminal and violent behaviors [3,5,9,21,22]. In addition, once the link with the experience of violence is established, the child may learn such behaviors and replicate them, perpetuating other types of violence such as child-to-parent violence [2]. With special interest in view of the results of a robust meta-analysis, it was found that child victims of exposure to violence were 71% more likely to be involved in the perpetuation of child-to-parent violence [23].

Child-to-parent violence (CPV) is a particular type of family violence that has generated many studies in the last 10 years and has gained popularity in some countries such as Spain, the United Kingdom, and the United States [24,25,26,27], especially because it has revealed a series of findings that have generated alarm and concern about its impact on family relationships [28,29]. According to some studies, victims mainly experience stress, isolation, self-blame, shame, and a devastating impact on family and work relationships [30,31,32]. CPV is characterized by a repeated pattern of violent behavior perpetrated by children [33,34], usually under 18 years of age, although the age may be extended depending on the age of emancipation [35]. Children exert violent behaviors of psychological, physical, and economic types by means of controlling behaviors [33,34,36]. One of the most debated issues in the various definitions of CPV is the difficulty of assessing the control/domain of a company’s CPV [33], but this aspect is key, since the aggressor tries to make the parents feel confused and lose control over themselves in order to cause consternation, prejudice, and pain in the victim [36].

Children who engage in CPV are motivated by a goal, for example, wanting to receive more money or more freedom for their activities (instrumental reasons, IRs). However, they may also act as a consequence or reaction to the violence experienced (reactive reasons, RRs), for example, if they have previously suffered humiliation or experienced some type of victimization [36,37,38]. In a study conducted by Contreras et al. [39], it was found that both reactive and instrumental reasons were variables that interacted as mediators between children who had been victims of violence and the subsequent commission of CPV. Along the same lines, other studies have focused on analyzing the profile of aggressors; the findings have revealed that aggressors who use mostly reactive violence and have been victimized directly tend to be specialist aggressors (they only use CPV), while aggressors who use reactive and instrumental violence tend to be generalist aggressors (they use CPV and other types of violence) [40,41]. In addition, a recent study found that CPV toward both parents that is motivated by reactive and instrumental reasons is common in all victimization experiences, except for indirect victimization of the father toward the mother during the last year, where reactive reasons are the most frequent [42].

Another issue that has not been fully resolved in research on exposure to violence and CPV is the role of the sex of the victims and perpetrators. It is not surprising that most studies have reported contradictory results [24,25,26]. Regarding exposure to violence, some studies have reported that men are more likely to engage in rough play, fights, carry weapons, and become involved in fights, which would explain why men suffer more victimization at school or on the streets. While men are taught to be tough and aggressive, women are expected to be more sensitive and maintain good relationships [43,44,45]. These differences are clearly related to gender norms learned through the processes of gender socialization [46].

Social learning theory has motivated the development of several studies to elucidate the link between exposure to violence and CPV based on the fact that violent behaviors can be learned in different contexts and can later be used in different life situations, such as for problem solving [47]. Along these lines, a number of studies have found that exposure to domestic violence is an important predictor variable for CPV [7,39,48,49]. However, Calvete [16] found that children with low levels of exposure to domestic violence showed high levels of severe psychological violence and low levels of physical violence. These findings place us before a reality that needs to be investigated to provide a concrete response.

Few studies have focused their attention on the effects of exposure to violence from different scenarios in the CPV. Exposure to violence may have a cumulative effect but be different depending on the context in which victimization occurs. Junco-Guerrero et al. [17] found a direct relationship between vicarious victimization and CPV, but this relationship was not found in the case of direct victimization at home and the mother’s CPV. In the case of the mother, direct victimization at home and the mother’s CPV were found to have a direct relationship, but in this case, no relationship was found between vicarious victimization and CPV. Contreras and Cano [50] found that both types of victimization are directly related to CPV.

Harris et al. [1] identified 10 measures to assess exposure to domestic violence. This systematic review concluded that the measures have strengths but do not have a standardized approach to assessing exposure to violence. In addition, only three measures have been used in low- and middle-income countries; thus, cross-cultural studies are scarce. It is noteworthy that the Violence Exposure Scale (VES) was not included in this systematic review [51], a scale that was created with a Spanish-speaking population and has demonstrated adequate reliability. In addition, this scale has been used in numerous studies that have focused on analyzing the link between exposure to violence and CPV [16,42,52,53].

### The Present Study

The present study focuses on analyzing the mediating role of children’s motivations in the relationship between having experienced violence as direct victimization and vicarious victimization at home, at school, on the street, and on TV and the subsequent perpetuation of CPV toward the father and toward the mother. Furthermore, in this study, we differentiated the timeframe between exposure to violence in the last year and during childhood (before the age of 10). Since the VES has not been previously validated and used in the Colombian population, our first objective was to analyze the psychometric properties, internal consistency, convergent and discriminant validity, and the invariance of the VES according to sex and age. The second objective was to analyze the differences in exposure to violence in different scenarios and CPV between boys and girls and younger and older adolescents. Finally, and as a third objective, we set out to examine the mediating role of IRs and RRs between exposure to violence during the last year and during childhood in different scenarios and CPV toward the fathers and toward the mothers.

The hypotheses of this study were as follows: (1) The VES will present a model with adequate psychometric properties to assess exposure to violence, in addition to strong evidence of validity, reliability, and invariance by sex and age; (2) there will be differences by sex and age according to the scenario and type of victimization; and (3) IRs and RRs will mediate the relationship between exposure to violence and CPV.

## 2. Materials and Methods

### 2.1. Participants

The sample consisted of 981 Colombian adolescents (51% female, 49% male) between 12 and 18 years of age (*M* = 14.53, *SD* = 1.55); 679 (70%) lived in Antioquia, and 290 (30%) lived in Magdalena. The socioeconomic stratum in Colombia is grouped into seven levels, with the proportion in our sample being 6 adolescents (0.7%) from stratum zero, 120 (15%) from stratum one, 283 (34%) from stratum two, 316 (38%) from stratum three, 74 (9%) from stratum four, 23 (3%) from stratum five, and 5 (0.6%) from stratum six.

### 2.2. Measures

Violence Exposure Scale (VES) [51]. This questionnaire was used to assess exposure to violence and was adapted to Colombia. The original version of the VES consists of 21 items assessing direct and vicarious victimization in four scenarios (home, street, school, and TV). The original version assessed only direct victimization and vicarious victimization in the last year. But in this study, an additional adaptation was made to assess exposure to violence in childhood.

Direct victimization in the home is assessed by three items (for example, *how often has your father hit or physically hurt you?*) and three items on vicarious victimization (for example, *how often have you seen your father hit or physically hurt your mother?*). Direct victimization at school was assessed similarly (for example, *how often have you been hit or physically hurt at school?*) and vicarious victimization in school (for example, *How often have you seen a person hit or physically harm another person at school?*). Exposure to violence on TV was assessed using three items (for example, *how often have you seen a person threaten to hit another person on TV?*). This instrument uses a 5-point Likert-type scale (0 = *never* to 4 = *every day*). The reliability of the VES factors in the initial study ranged from α of 0.71 to 0.80 [51].

Child-to-parent Violence Questionnaire (CPV-Q; adapted to the Colombian population) [54]. This scale was created by Contreras et al. [37] and is one of the most promising questionnaires to evaluate this type of family violence [27,55]. It consists of 14 parallel items for the father and the mother and evaluates the four typologies of CPV: (1) psychological CPV (for example, “*I have insulted my parents*”, *4 items*); (2) physical CPV (for example, “*I have thrown objects at my parents*”); (3) financial CPV (e.g., “*I stole money from my parents*”); (4) control/domain (for example, “*I have demanded that my parents do what I want at home*”). The temporality of the behaviors was measured for the last 12 months using a 5-point Likert-type scale (0 = *never* to 4 = *very often/six times or more*). The questions were answered separately by the fathers and mothers. The reliability of the father’s baseline CPV-Q measure ranged between α 0.74 and 0.75, and the mother’s ranged between α 0.76 and 0.77 [37].

This questionnaire includes a subscale to assess the reasons children engage in CPV behaviors. It consists of 8 parallel items for the father and mother. The reasons could be instrumental (for example, “*To avoid doing a chore*”) or reactive (e.g., “In *response to a previous physical assault by your parent*”). These items were evaluated using a four-point Likert-type scale (0 = never to 3 = always). The reasons were given separately according to the sex of the parents. The reliability of the original subscale was 0.74 for the instrumental reasons factor and 0.63 for the reactive reasons factor [37].

### 2.3. Procedure

This study was approved by the Ethics Committee of the Universidad San Buenaventura-Medellín (Resolution N° 1326, 14 de Agosto de 2023). The original version of the VES was based on studies with a Spanish-speaking population; however, this questionnaire has not been used with a Colombian population. To adapt the questionnaire to this population, we followed the PC-2, PC-3, and TD-1 guidelines from the International Test Commission [56]. First, we assessed the overlap between the definition and the content of the construct. At this stage, we verified that a recent systematic review identified 10 measures to assess exposure to child violence, all of which had been developed in the United States, the United Kingdom, and Canada and were rarely used in countries other than their environment [1]. This review did not include the VES as an instrument to assess exposure to violence; however, we verified that it was similar to the JVQ developed by Finklenhor et al. [57].

We decided to use the VES for the following reasons: (1) it has excellent psychometric properties and good reliability in the original version [51] in studies with Spanish-speaking populations [42,53,58]; (2) it is one of the few instruments that assesses exposure to violence in different settings, including exposure to violence on TV; and (3) this scale is the most widely used to study the relationship between exposure to violence and CPV [15,41,42,49,59]. We then interviewed experts on violence and asked them about the incidence of exposure to violence and CPV in Colombian children. Their answers confirmed the need to study this issue.

The next step was to elaborate on an adaptation of the timeframe of the VES. For this purpose, a parallel scale was added, called VES^1^, which evaluates exposure to violence in the last year, and VES^2^, which evaluates exposure to violence during childhood (before the age of 10). To minimize the influence of any cultural and linguistic differences in the adaptation, a focus group was formed with adolescents, and they were asked about their understanding of the instructions, comprehension difficulties regarding the items and their wording or expressions, their experience with psychological tests, and the time associated with the administration. They were also asked about possible difficulties associated with comprehension.

The subsequent stage of the process was to establish contact with four expert Colombian psychologists (who had extensive knowledge of culture) to review the VES, with the aim of considering aspects of culture, test content, and general test principles. The conclusion of the adolescent group and professionals was that the scale was fully understood and that there were no significant linguistic differences. Therefore, the ITC guidelines did not continue.

The undersecretariats of the Education of Antioquia and the directors of several schools were contacted to request authorization to carry out this research. Several schools in Antioquia and Magdalena participated in this study. The adolescents were first contacted in their school classrooms, informed of the objectives of the study and the ethical guarantees, and were given an informed consent form informing their legal guardians about the study, which they had to bring back signed by them within 72 h. Prior to the evaluation, participants were reminded of the ethical rules (anonymity, confidentiality, voluntary, and that they could withdraw at any time) and asked to sign an informed consent form.

The evaluations were group-based and carried out in the classrooms of the educational centers, with the application format being paper and pencil. The guided practice modality was followed during the evaluations, giving them the test, reading the test instructions, and explaining the Likert scale of the response. They first responded to the timeframe of the last year and then to the timeframe of childhood. An attempt was made to avoid variability in response times, once they had finished completing the items in the VES^1,2^ questionnaire of CPV (CPV-Q). The protocols were collected anonymously, and the confidentiality of the responses was guaranteed. The test administrators were researchers trained for the application of these instruments, and they were also trained to answer and resolve any doubts or act out any possible incidents of revictimization or psychological discomfort that might occur during the application. Finally, we thanked the participants, who did not receive any benefits, for their participation.

### 2.4. Data Analysis

Prior to all the analyses, the database was curated. Cases with high social desirability and inconsistent responses were eliminated; both procedures were carried out using 7 social desirability questions and an item included in the CPV-Q to control incongruent responses. Descriptive statistics (means and standard deviations) were calculated to summarize the participants’ characteristics. Multivariate normality was assessed using Mardia’s test, which showed that the data did not follow a multivariate normal distribution (Kurtosis VES^1^ = −0.206, *p* < 0.001; Kurtosis VES^2^ = −0.369, *p* < 0.001).

First, confirmatory factor analysis was used to determine the VES^1,2^. This analysis employed the diagonal weighted least squares (DWLSs) method for ordinal data, using a polychoric correlation matrix and an asymptotic covariance matrix. The criteria for evaluating the model were the criteria proposed by Brown [60] RMSEA < 0.05, SRMR < 0.080, CFI > 0.95, and TLI > 0.95. The factor loadings and residual matrix were analyzed to identify discrepancies. The criterion for detecting discrepancies in the residuals was >2. In this study, discrepancies were found between the factors direct and vicarious victimization at school and on the street.

The internal consistency of the scale and its factors was calculated using different coefficients: McDonald ordinal, CR, G.9, and Cronbach’s alpha ordinal. Although alpha is the most commonly used coefficient, its limitations led to the additional use of omega and CR, recommended for assessing internal consistency (minimum acceptable > 0.65) [35]. Convergent validity was determined by the mean variance extracted (AVE > 0.50) [61], and discriminant validity was determined with the HTMT (<0.85) index [62]. The psychometric properties of the CPV-Q and its subscale were also evaluated.

**Figure 1:**
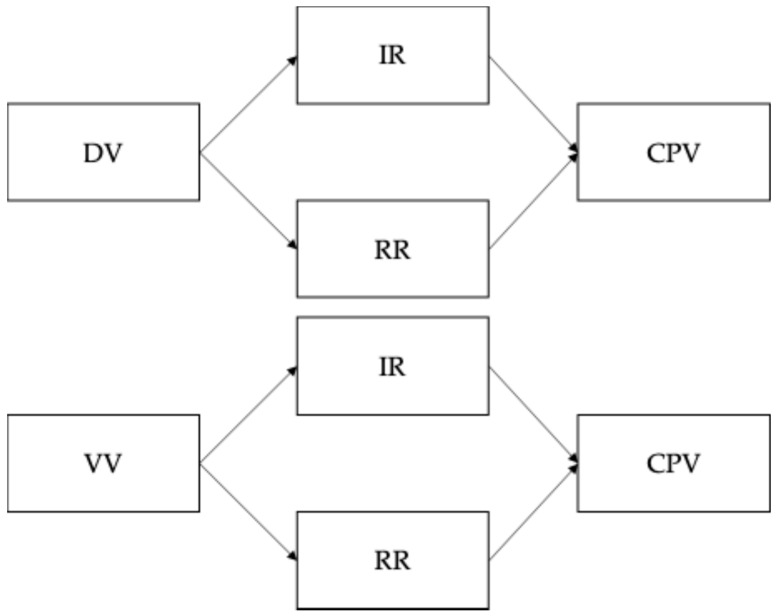
Mediating model between exposure to violence and child-to-parent violence. Note. DV = direct victimization at home, school, street, and TV; VV = vicarious victimization at home, school, and on the street; IRs = instrumental reasons; RRs = reactive reasons; CPV = child-to-parent violence.

The analysis of this objective involved evaluating the invariance of the VES^1,2^ according to sex (males and females) and age (12–14 years and 15–18 years), considering four levels: (1) configural invariance (equal factor structure); (2) metric invariance (equivalent factor loadings); (3) scalar invariance (equivalent intercepts); and (4) residual invariance (equivalent errors). Classical criteria were applied (CFI < 0.010; RMSEA < 0.015; SRMR < 0.025) to assume that the scale was invariant across the proposed groups [63]. To achieve the second objective, the Mann–Whitney U test was used to analyze differences by sex and age in direct and vicarious victimization in the four scenarios, as well as in CPV toward both parents, given that the data did not have a normal distribution. For the third objective, ten mediation model analyses were carried out with Hayes’ PROCESS macro (Model 4); four scenarios were analyzed (home, school, street and TV), where the independent variable was exposure to violence (last year and childhood), the mediators were the CPV motives (IRs and RRs), and the dependent variables were the CPV toward each parent (see Figure 1). A bootstrap was used with 5000 samples, and results with *p* < 0.05 and a confidence interval that did not include zero were considered significant [64]. The analyses were carried out with the R program (version 4.4.2) and JASP (version 0.19.2).

## 3. Results

We assessed the psychometric properties and internal consistency of the instruments used. For VES^1^ and VES^2^, adequate fit indices were reported, with satisfactory values in general. Both the scales showed solid internal consistency. As for the evidence of the validity of the CPVQ for fathers and mothers, acceptable fit indices and adequate internal consistency were obtained. The reason subscales present adequate adjustment indexes, although the RMSEA of both models presents slightly elevated values; in spite of this, the internal consistency coefficients indicate acceptable reliability for both subscales (see Table A2 in Appendix A).

The various internal consistency coefficients of the factors of the VES^1,2^: in all cases, an acceptable reliability was observed (ω ≥ 0.79; CR > 0.70). Next, the AVE of VES^1,2^ is presented, with a variance of 50% for each of the factors (AVE > 0.50). In the VES^1^, the factors of direct victimization in school with direct victimization on the street and vicarious victimization in school with vicarious victimization on the street had no discriminant validity; likewise, in the VES^2^, direct victimization in school with street victimization; vicarious victimization in school with vicarious victimization on the street; vicarious victimization in school with exposure to violence on TV; and vicarious victimization on the street with exposure to violence on TV had no discriminant validity (HTMT < 0.85, see Table A3 in Appendix A).

Tests of measurement invariance were conducted as a function of sex and age for the VES^3^ and VES^4^. Both models were tested under four added models of configural, metric, scalar, and strict invariance, with the benchmarks being satisfactory under all assumptions. The invariance of the VES^3^ by sex was M_C_ → M_M:_ ΔCFI = 0.002, ΔRMSEA = 0.003, ΔSRMR = 0.003; M_M_ → M_E:_ ΔCFI = 0.000, ΔRMSEA = 0.001, ΔSRMR = 0.000; M_E_ → M_S_: ΔCFI = 0.000, ΔRMSEA = 0.002, ΔSRMR = 0.002. The invariance of the VES^3^ by age was M_C_ → M_M:_ ΔCFI = 0.001, ΔRMSEA = 0.000, ΔSRMR = 0.001; M_M_ → M_E:_ ΔCFI = 0.001, ΔRMSEA = 0.001, ΔSRMR = 0.002; M_E_ → M_S_: ΔCFI = 0.001, ΔRMSEA = 0.001, ΔSRMR = 0.012. The invariance of the VES^4^ according to sex was M_C_ → M_M:_ ΔCFI = 0.001, ΔRMSEA = 0.002, ΔSRMR = 0.002; M_M_ → M_E_: ΔCFI = 0.001, ΔRMSEA = 0.002, ΔSRMR = 0.000; M_E_ → M_S_: ΔCFI = 0.000, ΔRMSEA = 0.003, ΔSRMR = 0.002. The invariance of the VES^4^ according to age was M_C_ → M_M:_ ΔCFI = 0.000, ΔRMSEA = 0.001, ΔSRMR = 0.002; M_M_ → M_E_: ΔCFI = 0.000, ΔRMSEA = 0.002, ΔSRMR = 0.001; M_E_ → M_S_: ΔCFI = 0.000, ΔRMSEA = 0.001, ΔSRMR = 0.001. See Table A4 in Appendix A.

Of the 981 participants, 94.8% reported having experienced at least some exposure to violence in one of the settings during the last year, and 87% during childhood. Table 1 presents the differences in exposure to violence by sex and age for both last year and childhood. In summary, it was found that during the last year, girls reported experiencing more direct victimization experiences at home, whereas boys reported higher levels of direct victimization at school and on the street. In childhood, boys reported experiencing higher rates of school and street victimization. With respect to age, older adolescents reported higher rates of victimization in general. During the last year, this group reported greater vicarious victimization at home, at school, and on TV; the same group reported experiencing greater direct victimization and vicarious victimization at home in addition to vicarious victimization at school, on the street, and on TV.

Table 2 shows the differences according to sex and age in CPV and the reasons for CPV of the father and mother. With respect to the fathers, girls were found to have higher RR scores, whereas with respect to the mother, girls reported higher CPV and RRs.

Table 3 shows that all models of direct and vicarious victimization with the father’s CPV were mediated by IRs and RRs in the last year. The effect of RRs was found to be greater than the effect of IRs in all models. In this timeframe, the exposure to home violence does not present variations with respect to exposure to direct and vicarious violence. Meanwhile, the coefficients on exposure to violence at school and on the street were higher when it came to exposure to direct violence.

Table 4 shows that all models of direct and vicarious victimization with the father’s CPV were mediated by IRs and RRs in childhood (before 10 years of age). In this timeframe, in the exposure to violence in the home, the coefficient of direct victimization was found to be higher than that of vicarious victimization. While at school, direct victimization was higher than vicarious victimization, and on the street, vicarious victimization was higher than direct victimization.

Variations also emerged in the effect of victimization by type of victimization and timeframe. Direct victimization in the past year had the greatest effect on the father’s CPV compared to vicarious victimization. In addition, it had a greater effect than victimizations in the other scenarios. In school, direct victimization was found to be higher than vicarious victimization in the last year and higher than direct and vicarious victimization that occurred during childhood; both direct and vicarious victimization presented similar effects in childhood. On the street, direct victimization had a greater effect than vicarious victimization, but childhood vicarious victimization was greater than direct victimization; also in this scenario, vicarious victimization in childhood had a greater effect overall. Exposure to TV violence was greater during childhood. The effect of RRs was found to be greater than the effect of IRs in all models.

Table 5 shows that all models of direct and vicarious victimization with the mother’s CPV were mediated by IRs and RRs in the last year. In this timeframe, it was found that in the model of exposure to violence in the home, vicarious victimization had a greater effect than direct victimization. At school and on the street, the effect of direct victimization was greater than that of vicarious victimization. 

Table 6 shows that all models of direct and vicarious victimization with the mother’s CPV were mediated by IRs and RRs in childhood (before 10 years age). In this timeframe, it was found that the coefficient of direct victimization in the home was higher than that of vicarious victimization, while in school and on the street, the coefficient of vicarious victimization was higher than that of direct victimization.

Variations emerged according to the type of victimization and timeframe. First, vicarious victimization in the home had a larger effect than direct victimization in the last year; direct victimization was greater than vicarious victimization in childhood, and vicarious victimization in this setting had a greater effect than victimization in the other settings. In school, direct victimization had a greater effect than vicarious victimization in the last year and vicarious victimization had a greater effect than direct victimization in childhood. On the street, direct victimization had a greater effect than vicarious victimization in the last year and vicarious victimization had a greater effect than direct victimization in childhood, and vicarious victimization in childhood had the greatest effect in this scenario. Exposure to TV violence had a greater effect during childhood. The effect of RRs was found to be greater than the effect of IRs in all models.

## 4. Discussion

In support of the first hypothesis, we found that the VES is an instrument that has strong evidence of convergent validity, the factors explained more than 50% of the variance, and the reliability of the overall scale and by factors was satisfactory. The psychometric model of the VES was acceptable for most of the fit indices. However, in the residual’s matrix, it was observed that the factors of vicarious victimization at school with the factor of vicarious victimization on the street showed discrepancies with the model. Therefore, it was necessary to correlate the errors of these two factors (school with street) to obtain an adequate RMSEA value. Error correlation is a procedure used when items of the factors share content and have similar formulations [65,66].

In this case, this practice is justified because the formulation of the VES items is similar; for example, one of the items to assess vicarious victimization at school was how often have you seen a person threatened with hitting another person at school, and for vicarious victimization on the street, the same item was used as the changed scenario, that is, how often have you seen a person threatened with hitting another person on the street? The first explanation is that by sharing a similar formulation, the errors of these two factors are correlated. Second, events experienced by adolescents at school may overlap with those occurring on the street because they may share experiences where violence co-occurs. A more conservative position recommends avoiding the use of this procedure or reflecting on the causes [67]. In this study, we found with further analysis that the VES model had excellent fit indices for the factors of exposure to violence at home, school, and on TV; therefore, in this case, there is no need to correlate the errors. In some studies, the VES has been used by subscales, such as exposure to violence at home [15,16,39,41,42]. Therefore, future studies should analyze the street factor separately. We suggest caution in interpreting these findings, at least until further studies can elucidate whether these findings are due to other factors such as participant fatigue or are a characteristic of this sample [67].

The discrepancies found in the correlation of the errors coincide with the problems in discriminant validity of the same factors; that is, direct and vicarious victimization at school share a high similarity with respect to the experiences of violence that co-occur in these scenarios. Both VES^1^ and VES^2^ were completely invariant to the sex and age of the adolescents, which has important implications for further differential analyses based on sex and age. These findings have important implications for the results obtained in the differences according to sex and age, since what is reported is due to the phenomenon and not to a bias of the instrument. These findings have the limitation of not being able to be contrasted with the previous literature given that, to our knowledge, this is the first study to report evidence of current validity and measures of invariance.

The second hypothesis was partially fulfilled because differences by sex and age were not found in all scenarios and timeframes. In the last year, girls reported experiencing more direct victimization at home, while boys reported more direct victimization at school and on the street. In childhood, boys reported experiencing more direct victimization at school and on the street. Other findings agree that the most relevant exposure to violence for boys occurs on the street, whereas for girls, it occurs in the home [52,68,69]. Although girls reported more victimization in their homes, this does not mean that they do not suffer victimization in other contexts; in fact, they are much more likely to suffer polyvictimization [68]. Therefore, it is of interest that future studies delve deeper into the factors underlying these differences. A possible explanation is that the type of socialization of boys may have a perspective of greater autonomy and therefore face a higher risk of victimization outside the home, that is, they may assume a style of instrumental violence, while that of girls may have a more relational character and be oriented to channel interpersonal relationships, which would explain the lower victimization in contexts outside the home [45]. In addition, these findings provide important information for intervention and violence prevention programs. As girls reported more victimization at home and boys reported more victimization at school and on the street, programs should incorporate a gender perspective, co-parenting, educational styles, and gender socialization processes [45] to mitigate exposure to violence and, where appropriate, sex differences among adolescents.

On the other hand, older adolescents reported experiencing more vicarious victimization in the home and on the street and on TV, while in childhood, it was found that older adolescents reported more direct and vicarious victimization in the home, in addition to experiencing vicarious victimization in schools and on the streets and on TV. This highlights that exposure to violence is widespread in the short and long term, so that once the link to violence is established, the spiral can repeat itself in adolescence. Furthermore, these findings are consistent with those reported by Finkelhor et al. [70], who found that older children (14–17 years) are more likely than younger children to be victims of physical and emotional abuse.

Regarding CPV, when directed toward the father, no differences were found according to adolescent sex; however, when CPV was directed toward the mother, it was found that girls reported higher scores and used more reactive reasons as motivations for exercising CPV toward mothers. This poses a complex reality, because, according to this study, the profile of the mother’s aggressors may be represented mainly by girls who, in turn, reported having been victims of exposure to violence in the home more than boys. In a sense, this would explain that if the parental role is mostly represented by the mother at home and rigid parental education and victimization is mainly directed toward girls, they would be carrying out CPV in response to previous aggressions.

It is also known that the profile of adolescent aggressors reported for assaulting a parent tends to be 60% between 14 and 17 years of age [71]; however, in this study, no differences were found according to the age of the adolescents who committed CPV. Although this finding is dissonant with other studies in which it has been reported that males who commit CPV are older than females, this does not seem to be the case [68]. It is not new to find that the results do not agree, as they will depend on many factors, such as the individual characteristics of the sample, the context, the culture, and other artifacts that may explain the variability of the results and even of the statistical analysis. Furthermore, these findings should be interpreted with caution until more information regarding these differences is available.

The third hypothesis was completely fulfilled since both instrumental and reactive reasons were mediating variables between exposure to violence and CPV. However, it is worth noting the variations according to the types of victimization, timeframe, and scenario of violence. In general, toward both parents, reactive reasons were found to have stronger effects than instrumental reasons in all scenarios and timeframes. This explains why adolescents who have experienced some adverse experiences, specifically exposure to violence, are more likely to commit CPV. This is congruent with other studies where reactive reasons were found to have a stronger effect than instrumental reasons in all scenarios and timeframes [42].

To explain fathers’ CPV, the two types of victimization were found to have significant effects [42], but direct victimization had a greater effect than vicarious victimization in both timeframes. This is consistent with the findings of the study of Navas et al. [49], where direct victimization was the best predictor of CPV toward the father, followed by vicarious victimization. In school, direct victimization had a greater effect than vicarious victimization in the last year, and in childhood, the effects of both types of victimization were similar. As in other studies, this predictor is the second most important predictor of CPV [49]. On the street, direct victimization was greater in the last year, and vicarious victimization had a greater effect in childhood. Exposure to violence on TV was higher in childhood. Therefore, the conclusion is that direct victimization in the last year of the three scenarios was the best predictor for CPV, highlighting that direct victimization in the home is the strongest predictor for father CPV.

With respect to mothers, vicarious victimization in the home had a greater effect in the last year, but direct victimization had a greater effect in childhood. Although the variations in the effect are small, this finding is of interest because it differs from the father’s CPV; that is, for the father, direct victimization has a greater effect on the mother’s vicarious victimization, which may be due to the fact that the exposure to violence that parents cause to their children is different according to the sex of the parents. At school, direct victimization had a greater effect than vicarious victimization in the last year, and vicarious victimization had a greater effect in childhood. On the street, direct victimization had a greater effect in the last year, and vicarious victimization had a greater effect in childhood. Exposure to violence on TV had a greater effect in childhood than in the last year.

### Limitations

The present study has a number of limitations that need to be resolved in further studies. First, a psychometric limitation is that we had to correlate the errors of the factors of exposure to violence at school with those of the street, and in spite of this, the criterion validity of these factors was critical. However, we present as an alternative using this scale with three factors, i.e., treating the street factor as an independent factor, at least until future studies allow us to elucidate the cause of psychometric problems. Secondly, we did not analyze variables that could be beneficial to deepen the understanding of the CPV, for example, the marital status of the parents, socioeconomic income, and the level of study of the parents. Therefore, these future studies should analyze the differential role of aspects such as socioeconomic status and thus approach a generalization of the results in the study samples.

The results of this study do not imply causality, as the data are cross-sectional and may contain some level of bias since they come from self-reports and were not contrasted with information from parents. Therefore, future studies should address the parents’ perspective and longitudinal studies. Variations were found according to the temporality of the coefficients, but they were minimal, so this study does not allow us to be conclusive about the differences in the cumulative effects by timeframe of exposure to violence. Finally, although the participants in this study belong to different geographical areas of Colombia, the sampling was intentional and not probabilistic, so the results are not generalizable to the population of Colombian adolescents, but they do allow us to have an idea of reality through the samples obtained.

Future studies should address the role of current technologies, social networks, and video games as means of victimization. Understanding the role of these new technologies will allow us to expand our knowledge, since TV may be a medium that is consumed less and less by adolescents. Despite these limitations, this study has certain strengths and implications for practices: (1) practitioners can use the VES to assess exposure to violence in three settings, considering the limitations of the school and street factors; (2) violence prevention interventions should focus first on family violence, though they should be careful to not neglect school-based interventions and awareness of violent content consumed through TV.

## Figures and Tables

**Table 1:** Differences between exposure to violence according to sex and age.

		Last Year (n = 981)				Childhood (Before 10 Years of Age) (n = 981)			
	G	*M* (*SD*)	*U*	*p*	*R-B C*	*M* (*SD*)	*U*	*p*	*R-B C*
**Sex**									
DV home	B	3.300 (3.655)	107,220.000	0.003	−0.109	3.688 (3.896)	117,551.500	0.529	−0.023
	G	3.948 (3.763)				3.934 (4.055)			
VV home	B	2.587 (3.441)	113,676.000	0.120	−0.055	2.988 (3.901)	112,764.000	0.075	−0.062
	G	2.944 (3.669)				3.487 (4.212)			
DV school	B	2.401 (2.396)	137,627.000	<0.001	0.144	1.878 (2.407)	135,808.500	<0.001	0.129
	G	1.881 (2.309)				1.427 (2.272)			
VV school	B	4.917 (3.431)	127,055.500	0.125	0.056	3.318 (3.242)	123,333.000	0.483	0.025
	G	4.598 (3.040)				3.091 (2.988)			
DV street	B	1.749 (2.368)	137,457.000	<0.001	0.143	1.287 (2.102)	131,771.500	0.002	0.096
	G	1.135 (1.883)				0.901 (1.788)			
VV street	B	5.066 (3.184)	121,858.500	0.720	0.013	3.467 (3.288)	119,946.000	0.940	−0.003
	G	5.034 (3.102)				3.423 (3.129)			
TV	B	5.671 (3.669)	119,678.000	0.893	−0.005	4.045 (3.615)	121,130.000	0.845	0.007
	G	5.805 (3.722)				3.974 (3.494)			
**Age**									
DV home	G1	3.579 (3.704)	117,918.500	0.590	−0.020	3.287 (3.824)	101,249.000	<0.001	−0.158
	G2	3.687 (3.744)				4.354 (4.063)			
VV home	G1	2.538 (3.458)	110,193.000	0.018	−0.084	2.845 (3.908)	105,682.500	<0.001	−0.121
	G2	3.014 (3.652)				3.648 (4.190)			
DV school	G1	2.263 (2.451)	126,467.500	0.152	−0.052	1.528 (2.209)	114,141.500	0.138	−0.051
	G2	2.008 (2.268)				1.774 (2.481)			
VV school	G1	4.584 (3.149)	113,961.500	0.153	−0.052	2.928 (3.022)	108,342.000	0.006	−0.099
	G2	4.932 (3.327)				3.487 (3.190)			
DV street	G1	1.335 (2.058)	114,425.500	0.151	−0.049	0.974 (1.810)	114,217.000	0.110	−0.050
	G2	1.534 (2.251)				1.213 (2.094)			
VV street	G1	4.711 (3.207)	104,600.500	<0.001	0.130	2.986 (3.064)	99,850.000	<0.001	−0.170
	G2	5.400 (3.036)				3.917 (3.285)			
TV	G1	5.412 (3.622)	108,223.000	0.006	−0.100	3.586 (3.495)	102,441.500	<0.001	−0.148
	G2	6.077 (3.741)				4.445 (3.563)			

Note. B = boys; G: girls; G1 = adolescents 12–14 years; G2 = adolescents 15–18 years; R-B C = Rank–Biserial Correlation; DV = direct victimization; VV = vicarious victimization; U = Mann–Whitney U test.

**Table 2:** Sex and age differences in child-to-parent violence and the reasons for it.

		Father (n = 887)				Mother (n = 887)			
		*M* (*SD*)	*U*	*p*	*R-B C*	*M* (*SD*)	*U*	*p*	*R-B C*
**Sex**									
CPV	B	4.314 (6.184)	73,165.000	0.114	−0.065	4.172 (5.719)	66,813.500	<0.001	−0.156
	G	4.205 (3.972)				4.553 (3.947)			
IRs	B	1.795 (2.501)	84,130.000	0.050	0.076	1.873 (2.384)	83,642.500	0.076	0.069
	G	1.412 (2.026)				1.520 (1.932)			
RRs	B	0.944 (1.688)	71,235.000	0.016	−0.089	0.939 (1.661)	66,647.000	<0.001	−0.148
	G	1.078 (1.625)				1.235 (1.680)			
**Age**									
CPV	G1	3.798 (3.918)	67,773.500	0.060	−0.078	4.059 (3.947)	70,275.000	0.290	−0.044
	G2	4.579 (5.768)				4.595 (5.267)			
IRs	G1	1.587 (2.189)	74,424.000	0.749	0.000	1.707 (2.170)	74,172.000	0.819	0.009
	G2	1.627 (2.400)				1.691 (2.191)			
RRs	G1	0.913 (1.487)	71,094.500	0.386	−0.033	1.082 (1.672)	73,016.000	0.864	−0.007
	G2	1.112 (1.816)				1.096 (1.684)			

Note. B = boys; G: girls; G1 = adolescents 12–14 years; G2 = adolescents 15–18 years; R-B C = Rank–Biserial Correlation; CPV = child-to-parent violence; IRs = instrumental reasons; RRs = reactive reasons; U = Mann–Whitney U test.

**Table 3:** Models between exposure to violence in different contexts and child-to-parent violence toward the father mediated by instrumental and reactive reasons in the last year.

	Last Year (n = 791)								
	Nonstandard Coefficient	Standard Coefficient	LLCI-ULCI	*p*		Nonstandard Coefficient	Standard Coefficient	LLCI-ULCI	*p*
Home	Dependent variable: CPV (R2 = 0.443), Mediators: IR (R2 = 0.060), RR (R2 = 0.137)				Dependent variable: CPV (R2 = 0.444), Mediators: IR (R2 = 0.049), RR (R2 = 0.116)				
DV → IR	0.153 (0.021)	0.248 (0.039)	0.170–0.323	<0.001	VV → IR	0.143 (0.029)	0.221 (0.041)	0.140–0.301	<0.001
DV → RR	0.166 (0.015)	0.371 (0.034)	0.299–0.436	<0.001	VV → RR	0.160 (0.022)	0.340 (0.041)	0.260–0.419	<0.001
IR → CPV	0.748 (0.068)	0.329 (0.054)	0.216–0.429	<0.001	IR → CPV	0.759 (0.132)	0.329 (0.053)	0.218–0.428	<0.001
RR → CPV	1.319 (0.098)	0.421 (0.050)	0.330–0.527	<0.001	RR → CPV	1.301 (0.217)	0.421 (0.051)	0.324–0.521	<0.001
DV → RI → CPV	0.114 (0.019)	0.082 (0.018)	0.049–0.122	<0.001	VV → RI → CPV	0.107 (0.019)	0.073 (0.018)	0.042–0.112	<0.001
DV → RR → CPV	0.219 (0.025)	0.156 (0.025)	0.114–0.211	<0.001	VV → RR → CPV	0.211 (0.026)	0.143 (0.023)	0.104–0.192	<0.001
DV → CPV	0.418 (0.048)	0.298 (0.039)	0.215–0.367	<0.001	VV → CPV	0.415 (0.050)	0.282 (0.046)	0.185–0.367	<0.001
School	Dependent variable: CPV (R2 = 0.445), Mediators: IR (R2 = 0.039), RR (R2 = 0.066)				Dependent variable: CPV (R2 = 0.443), Mediators: IR (R2 = 0.007), RR (R2 = 0.029)				
DV → IR	0.196 (0.034)	0.201 (0.042)	0.120–0.423	<0.001	VV → IR	0.062 (0.029)	0.088 (0.039)	0.015–0.165	<0.001
DV → RR	0.184 (0.024)	0.259 (0.042)	0.175–0.341	<0.001	VV → RR	0.089 (0.024)	0.175 (0.038)	0.098–0.247	<0.001
IR → CPV	0.744 (0.068)	0.327 (0.053)	0.219–0.423	<0.001	IR → CPV	0.760 (0.129)	0.334 (0.054)	0.223–0.429	<0.001
RR → CPV	1.332 (0.095)	0.425 (0.047)	0.338–0.518	<0.001	RR → CPV	1.352 (0.210)	0.432 (0.047)	0.346–0.529	<0.001
DV → RI → CPV	0.146 (0.029)	0.066 (0.006)	0.015–0.049	<0.001	VV → RI → CPV	0.047 (0.019)	0.029 (0.014)	0.006–0.063	0.037
DV → RR → CPV	0.245 (0.037)	0.110 (0.007)	0.027–0.075	<0.001	VV → RR → CPV	0.120 (0.026)	0.075 (0.019)	0.041–0.115	<0.001
DV → CPV	0.554 (0.076)	0.250 (0.015)	0.064–0.162	<0.001	VV → CPV	0.253 (0.056)	0.158 (0.036)	0.081–0.226	<0.001
Street	Dependent variable: CPV (R2 = 0.441), Mediators: IR (R2 = 0.030), RR (R2 = 0.039)				Dependent variable: CPV (R2 = 0.440), Mediators: IR (R2 = 0.027), RR (R2 = 0.029)				
DV → IR	0.186 (0.052)	0.177 (0.048)	0.088–0.275	<0.001	VV → IR	0.123 (0.029)	0.168 (0.039)	0.086–0.243	<0.001
DV → RR	0.153 (0.034)	0.201 (0.043)	0.116–0.286	<0.001	VV → RR	0.093 (0.020)	0.175 (0.036)	0.103–0.242	<0.001
IR → CPV	0.752 (0.126)	0.331 (0.054)	0.211–0.429	<0.001	IR → CPV	0.756 (0.130)	0.332 (0.053)	0.221–0.428	<0.001
RR → CPV	1.363 (0.207)	0.435 (0.049)	0.348–0.539	<0.001	RR → CPV	1.373 (0.211)	0.438 (0.048)	0.352–0.539	<0.001
DV → RI → CPV	0.140 (0.030)	0.059 (0.019)	0.027–0.103	0.002	VV → RI → CPV	0.093 (0.093)	0.056 (0.016)	0.029–0.091	<0.001
DV → RR → CPV	0.209 (0.039)	0.087 (0.022)	0.048–0.135	<0.001	VV → RR → CPV	0.127 (0.127)	0.077 (0.017)	0.045–0.114	<0.001
DV → CPV	0.439 (0.084)	0.184 (0.051)	0.091–0.291	<0.001	VV → CPV	0.254 (0.058)	0.153 (0.037)	0.082–0.181	<0.001
TV	Dependent variable: CPV in TV (R2 = 0.443), Mediators: IR (R2 = 0.002), RR (R2 = 0.021)								
TV → IR	0.060 (0.022)	0.060 (0.039)	−0.017–0.179	0.124					
TV → RR	0.149 (0.016)	0.149 (0.034)	0.080–0.217	<0.001					
IR → CPV	0.335 (0.068)	0.335 (0.053)	0.223–0.434	<0.001					
RR → CPV	1.342 (0.094)	0.430 (0.048)	0.345–0.531	<0.001					
TV → IR → CPV	0.028 (0.017)	0.020 (0.014)	−0.005–0.049	0.138					
TV → RR → CPV	0.090 (0.022)	0.064 (0.016)	0.035–0.100	<0.001					
TV → CPV	0.215 (0.049)	0.153 (0.034)	0.082–0.219	<0.001					

Note. DV = direct victimization; VV = vicarious victimization; IRs = instrumental reasons; RRs = reactive reasons; CPV = child-to-parent violence; LLCI = lower limit confidence interval; ULCI = upper limit confidence interval.

**Table 4:** Models between exposure to violence in different contexts and child-to-parent violence toward the father mediated by instrumental and reactive reasons in childhood.

	Childhood (Before 10 Years of Age) (n = 791)								
	Nonstandard Coefficient	Standard Coefficient	LLCI-ULCI	*p*		Nonstandard Coefficient	Standard Coefficient	LLCI-ULCI	*p*
Home	Dependent variable: CPV (R^2^ = 0.445), Mediators: IR (R^2^ = 0.026), RR (R^2^ = 0.117)				Dependent variable: CPV (R2 = 0.446), Mediators: IR (R2 = 0.020), RR (R2 = 0.077)				
DV → IR	0.096 (0.029)	0.166 (0.040)	0.084–0.244	<0.001	VV → IR	0.082 (0.020)	0.145 (0.009)	0.068–0.223	<0.001
DV → RR	0.143 (0.022)	0.343 (0.037)	0.267–0.412	<0.001	VV → RR	0.115(0.014)	0.280 (0.008)	0.198–0.357	<0.001
IR → CPV	0.759 (0.132)	0.334 (0.053)	0.224–0.431	<0.001	IR → CPV	0.757 (0.068)	0.333 (0.030)	0.223–0.432	<0.001
RR → CPV	1.301 (0.217)	0.415 (0.050)	0.328–0.524	<0.001	RR → CPV	1.311 (0.096)	0.418 (0.031)	0.332–0.517	<0.001
DV → RI → CPV	0.073 (0.019)	0.056 (0.016)	0.027–0.091	<0.001	VV → RI → CPV	0.062 (0.016)	0.048 (0.015)	0.022–0.084	0.022
DV → RR → CPV	0.187 (0.026)	0.143 (0.024)	0.101–0.195	<0.001	VV → RR → CPV	0.151 (0.021)	0.117 (0.023)	0.077–0.169	<0.001
DV → CPV	0.358 (0.050)	0.273 (0.037)	0.195–0.341	<0.001	VV → CPV	0.219 (0.044)	0.248 (0.039)	0.110–0.320	<0.001
School	Dependent variable: CPV (R2 = 0.444), Mediators: IR (R2 = 0.021), RR (R2 = 0.039)				Dependent variable: CPV (R2 = 0.447), Mediators: IR (R2 = 0.008), RR (R2 = 0.037)				
DV → IR	0.142 (0.035)	0.144 (0.043)	0.062–0.231	<0.001	VV → IR	0.065 (0.026)	0.088 (0.040)	0.010–0.168	0.032
DV → RR	0.142 (0.035)	0.198 (0.041)	0.117–0.281	<0.001	VV → RR	0.104 (0.019)	0.193 (0.041)	0.117–0.274	<0.001
IR → CPV	0.751 (0.068)	0.330 (0.054)	0.219–0.426	<0.001	IR → CPV	0.761 (0.068)	0.335 (0.053)	0.223–0.430	<0.001
RR → CPV	1.347 (0.094)	0.430 (0.048)	0.344–0.532	<0.001	RR → CPV	1.330 (0.094)	0.424 (0.046)	0.223–0.430	<0.001
DV → RI → CPV	0.107 (0.028)	0.048 (0.017)	0.019–0.086	0.005	VV → RI → CPV	0.049 (0.021)	0.029 (0.015)	0.005–0.063	0.048
DV → RR → CPV	0.191 (0.036)	0.085 (0.020)	0.050–0.130	<0.001	VV → RR → CPV	0.138 (0.027)	0.082 (0.020)	0.047–0.129	<0.001
DV → CPV	0.444 (0.078)	0.197 (0.045)	0.117–0.294	<0.001	VV → CPV	0.330 (0.059)	0.195 (0.041)	0.115–0.276	<0.001
Street	Dependent variable: CPV (R2 = 0.442), Mediators: IR (R2 = 0.026), RR (R2 = 0.045)				Dependent variable: CPV (R2 = 0.440), Mediators: IR (R2 = 0.027), RR (R2 = 0.029)				
DV → IR	0.226 (0.059)	0.197 (0.050)	0.103–0.298	<0.001	VV → IR	0.121 (0.033)	0.166 (0.042)	0.088–0.275	<0.001
DV → RR	0.178 (0.036)	0.214 (0.042)	0.130–0.294	<0.001	VV → RR	0.111 (0.024)	0.210 (0.041)	0.116–0.286	<0.001
IR → CPV	0.749 (0.127)	0.329 (0.053)	0.222–0.427	<0.001	IR → CPV	0.747 (0.131)	0.328 (0.05)	0.211–0.429	<0.001
RR → CPV	1.359 (0.207)	0.434 (0.048)	0.343–0.533	<0.001	RR → CPV	1.343 (0.204)	0.429 (0.049)	0.348–0.539	<0.001
DV → RI → CPV	0.169 (0.034)	0.065 (0.020)	0.032–0.111	0.001	VV → RI → CPV	0.090 (0.021)	0.059 (0.019)	0.027–0.103	0.001
DV → RR → CPV	0.242 (0.043)	0.093 (0.020)	0.057–0.137	<0.001	VV → RR → CPV	0.149 (0.027)	0.087 (0.022)	0.048–0.135	<0.001
DV → CPV	0.527 (0.091)	0.202 (0.059)	0.094–0.324	<0.001	VV → CPV	0.357 (0.057)	0.216 (0.042)	0.129–0.295	<0.001
TV	Dependent variable: CPV (R2 = 0.443), Mediators: IR (R2 = 0.010), RR (R2 = 0.051)								
TV → IR	0.065 (0.023)	0.101 (0.040)	0.021–0.177	0.012					
TV → RR	0.106 (0.016)	0.226 (0.039)	0.146–0.300	<0.001					
IR → CPV	0.762 (0.068)	0.335 (0.052)	0.226–0.432	<0.001					
RR → CPV	1.342 (0.095)	0.428 (0.047)	0.339–0.525	<0.001					
TV → IR → CPV	0.049 (0.018)	0.034 (0.015)	0.008–0.066	0.022					
TV → RR → CPV	0.142 (0.024)	0.097 (0.021)	0.060–0.144	<0.001					
TV → CPV	0.272 (0.051)	0.185 (0.041)	0.100–0.264	<0.001					

Note. DV = direct victimization; VV = vicarious victimization; IRs = instrumental reasons; RRs = reactive reasons; CPV = child-to-parent violence; LLCI = lower limit confidence interval; ULCI = upper limit confidence interval.

**Table 5:** Results of mediation models reporting the mediating effects of instrumental and reactive reasons on the association of exposure to violence and child-to-parent violence toward the father.

	Last Year (n = 791)								
	Nonstandard Coefficient	Standard Coefficient	LLCI-ULCI	*p*		Nonstandard Coefficient	Standard Coefficient	LLCI-ULCI	*p*
Home	Dependent variable: CPV (R2 = 0.373), Mediators: IR (R2 = 0.037), RR (R2 = 0.113)				Dependent variable: CPV (R2 = 0.373); Mediators: IR (R2 = 0.045), RR (R2 = 0.134)				
DV → IR	0.159 (0.024)	0.271 (0.037)	0.199–0.344	<0.001	VV → IR	0.131 (0.021)	0.212 (0.041)	0.131–0.293	<0.001
DV → RR	0.199 (0.020)	0.439 (0.031)	0.375–0.495	<0.001	VV → RR	0.174 (0.016)	0.366 (0.041)	0.286–0.445	<0.001
IR → CPV	0.543 (0.108)	0.240 (0.048)	0.145–0.333	<0.001	IR → CPV	0.551 (0.070)	0.244 (0.048)	0.147–0.332	<0.001
RR → CPV	1.227 (0.217)	0.419 (0.053)	0.319–0.525	<0.001	RR → CPV	1.245(0.095)	0.425 (0.052)	0.326–0.526	<0.001
DV → RI → CPV	0.086 (0.016)	0.065 (0.016)	0.038–0.100	<0.001	VV → RI → CPV	0.072 (0.015)	0.052 (0.014)	0.027–0.083	<0.001
DV → RR → CPV	0.244 (0.026)	0.184 (0.027)	0.134–0.241	<0.001	VV → RR → CPV	0.217 (0.026)	0.155(0.026)	0.110–0.213	<0.001
DV → CPV	0.460 (0.044)	0.249 (0.035)	0.275–0.414	<0.001	VV → CPV	0.424 (0.047)	0.304 (0.044)	0.209–0.381	<0.001
School	Dependent variable: CPV (R2 = 0.368), Mediators: IR (R2 = 0.063), RR (R2 = 0.102)				Dependent variable: CPV (R2 = 0.375), Mediators: IR (R2 = 0.015), RR (R2 = 0.041)				
DV → IR	0.234 (0.032)	0.251 (0.037)	0.179–0.324	<0.001	VV → IR	0.083 (0.027)	0.123 (0.038)	0.046–0.195	0.001
DV → RR	0.228 (0.024)	0.319 (0.042)	0.232–0.399	<0.001	VV → RR	0.110 (0.022)	0.202 (0.036)	0.130–0.273	<0.001
IR → CPV	0.545 (0.070)	0.241 (0.049)	0.142–0.334	<0.001	IR → CPV	0.557 (0.105)	0.247 (0.048)	0.151–0.327	<0.001
RR → CPV	1.289 (0.093)	0.440 (0.046)	0.350–0.529	<0.001	RR → CPV	1.283 (0.192)	0.446 (0.046)	0.358–0.524	<0.001
DV → RI → CPV	0.127 (0.024)	0.061 (0.016)	0.033–0.094	<0.001	VV → RI → CPV	0.046 (0.015)	0.031 (0.011)	0.010–0.055	0.010
DV → RR → CPV	0.294 (0.038)	0.140 (0.025)	0.095–0.194	<0.001	VV → RR → CPV	0.141 (0.026)	0.090 (0.020)	0.053–0.131	<0.001
DV → CPV	0.563 (0.072)	0.268 (0.043)	0.187–0.352	<0.001	VV → CPV	0.355 (0.055)	0.183 (0.036)	0.116–0.246	<0.001
Street	Dependent variable: CPV (R2 = 0.366), Mediators: IR (R2 = 0.051), RR (R2 = 0.047)				Dependent variable: CPV (R2 = 0.365), Mediators: IR (R2 = 0.048), RR (R2 = 0.040)				
DV → IR	0.250 (0.055)	0.229 (0.048)	0.134–0.325	<0.001	VV → IR	0.154 (0.027)	0.222 (0.037)	0.148–0.337	<0.001
DV → RR	0.185 (0.036)	0.220 (0.046)	0.138–0.308	<0.001	VV → RR	0.108 (0.019)	0.202 (0.032)	0.137–0.265	<0.001
IR → CPV	0.553 (0.103)	0.245 (0.049)	0.148–0.338	<0.001	IR → CPV	0.555 (0.104)	0.246 (0.047)	0.154–0.337	<0.001
RR → CPV	1.323 (0.195)	0.451 (0.046)	0.363–0.544	<0.001	RR → CPV	1.327 (0.201)	0.453 (0.046)	0.366–0.546	<0.001
DV → RI → CPV	0.139 (0.027)	0.056 (0.015)	0.029–0.090	<0.001	VV → RI → CPV	0.086 (0.017)	0.055 (0.010)	0.030–0.087	<0.001
DV → RR → CPV	0.245 (0.042)	0.099 (0.022)	0.061–0.148	<0.001	VV → RR → CPV	0.144 (0.027)	0.096 (0.018)	0.058–0.128	<0.001
DV → CPV	0.476 (0.086)	0.193 (0.063)	0.082–0.332	<0.001	VV → CPV	0.281 (0.055)	0.179 (0.032)	0.113–0.241	<0.001
TV	Dependent variable: CPV (R2 = 0.375), Mediators: IR (R2 = 0.007), RR (R2 = 0.022)								
TV → IR	0.051 (0.023)	0.086 (0.038)	0.156–0.340	0.022					
TV → RR	0.068 (0.017)	0.149 (0.033)	0.086–0.214	<0.001					
IR → CPV	0.560 (0.107)	0.248 (0.047)	0.159–0.340	<0.001					
RR → CPV	1.300 (0.202)	0.444 (0.046)	0.356–0.537	<0.001					
TV → IR → CPV	0.028 (0.012)	0.021 (0.010)	0.004–0.046	0.042					
TV → RR → CPV	0.090 (0.022)	0.066 (0.016)	0.037–0.100	<0.001					
TV → CPV	0.248 (0.046)	0.187 (0.030)	0.129–0.246	<0.001					

Note. DV = direct victimization; VV = vicarious victimization; IRs = instrumental reasons; RRs = reactive reasons; CPV = child-to-parent violence; LLCI = lower limit confidence interval; ULCI = upper limit confidence interval.

**Table 6:** Results of mediation models reporting the mediating effects of instrumental and reactive reasons on the association of exposure to violence and child-to-parent violence toward the mother.

	Childhood (Before 10 Years of Age) (n = 791)								
	Nonstandard Coefficient	Standard Coefficient	LLCI-ULCI	*p*		Nonstandard Coefficient	Standard Coefficient	LLCI-ULCI	*p*
Home	Dependent variable: CPV (R2 = 0.373), Mediators: IR (R2 = 0.038), RR (R2 = 0.112)				Dependent variable: CPV (R2 = 0.371), Mediators: IR (R2 = 0.022), RR (R2 = 0.091)				
DV → IR	0.107 (0.023)	0.195 (0.038)	0.116–0.266	<0.001	VV → IR	0.081 (0.022)	0.151 (0.039)	0.075–0.229	<0.001
DV → RR	0.142 (0.018)	0.337 (0.034)	0.268–0.403	<0.001	VV → RR	0.126 (0.019)	0.304 (0.036)	0.227–0.381	<0.001
IR → CPV	0.552 (0.108)	0.244 (0.047)	0.152–0.338	<0.001	IR → CPV	0.561 (0.108)	0.248 (0.047)	0.151–0.338	<0.001
RR → CPV	1.251 (0.195)	0.427 (0.048)	0.333–0.520	<0.001	RR → CPV	1.269 (0.194)	0.433 (0.056)	0.341–0.522	<0.001
DV → RI → CPV	0.059 (0.013)	0.048 (0.013)	0.026–0.076	<0.001	VV → RI → CPV	0.046 (0.012)	0.038 (0.012)	0.018–0.064	0.001
DV → RR → CPV	0.178 (0.022)	0.144 (0.023)	0.103–0.192	<0.001	VV → RR → CPV	0.160 (0.021)	0.132 (0.024)	0.091–0.184	<0.001
DV → CPV	0.359 (0.042)	0.290 (0.031)	0.227–0.350	<0.001	VV → CPV	0.308 (0.042)	0.253 (0.035)	0.181–0.320	<0.001
School	Dependent variable: CPV (R2 = 0.368), Mediators: IR (R2 = 0.030), RR (R2 = 0.051)				Dependent variable: CPV (R2 = 0.376), Mediators: IR (R2 = 0.014), RR (R2 = 0.041)				
DV → IR	0.163 (0.033)	0.173 (0.040)	0.094–0.252	<0.001	VV → IR	0.083 (0.025)	0.117 (0.038)	0.014–0.062	0.002
DV → RR	0.165 (0.025)	0.227 (0.039)	0.151–0.304	<0.001	VV → RR	0.110 (0.019)	0.202 (0.037)	0.040–0.093	<0.001
IR → CPV	0.554 (0.070)	0.245 (0.047)	0.149–0.331	<0.001	IR → CPV	0.557 (0.069)	0.247 (0.048)	0.156–0.336	<0.001
RR → CPV	1.307 (0.092)	0.446 (0.046)	0.358–0.540	<0.001	RR → CPV	1.283 (0.091)	0.438 (0.044)	0.328–0.580	<0.001
DV → RI → CPV	0.091 (0.022)	0.043 (0.013)	0.020–0.071	<0.001	VV → RI → CPV	0.046 (0.015)	0.029 (0.011)	0.009–0.055	0.012
DV → RR → CPV	0.215 (0.036)	0.101 (0.021)	0.062–0.145	<0.001	VV → RR → CPV	0.141 (0.026)	0.088 (0.020)	0.053–0.131	<0.001
DV → CPV	0.432 (0.074)	0.203 (0.048)	0.118–0.302	<0.001	VV → CPV	0.355 (0.055)	**0.222 (0.037)**	0.149–0.294	<0.001
Street	Dependent variable: CPV (R2 = 0.336), Mediators: IR (R2 = 0.051), RR (R2 = 0.047)				Dependent variable: CPV (R2 = 0.376), Mediators: IR (R2 = 0.036), RR (R2 = 0.042)				
DV → IR	0.250 (0.056)	0.229 (0.048)	0.140–0.329	<0.001	VV → IR	0.131 (0.048)	0.189 (0.039)	0.110–0.265	<0.001
DV → RR	0.185 (0.036)	0.220 (0.042)	0.141–0.307	<0.001	VV → RR	0.109 (0.042)	0.204 (0.038)	0.125–0.274	<0.001
IR → CPV	0.553 (0.106)	0.244 (0.048)	0.144–0.336	<0.001	IR → CPV	0.537 (0.048)	0.238 (0.047)	0.139–0.327	<0.001
RR → CPV	1.323 (0.196)	0.451 (0.046)	0.363–0.542	<0.001	RR → CPV	1.293 (0.046)	0.441 (0.045)	0.356–0.531	<0.001
DV → RI → CPV	0.139 (0.027)	0.056 (0.015)	0.030–0.090	<0.001	VV → RI → CPV	0.070 (0.015)	0.045 (0.013)	0.022–0.075	<0.001
DV → RR → CPV	0.245 (0.042)	0.099 (0.022)	0.061–0.147	<0.001	VV → RR → CPV	0.141 (0.022)	0.090 (0.020)	0.053–0.133	<0.001
DV → CPV	0.476 (0.086)	0.193 (0.064)	0.086–0.337	<0.001	VV → CPV	0.377 (0.064)	**0.241 (0.039)**	0.160–0.314	<0.001
TV	Dependent variable: CPV (R2 = 0.375), Mediators: IR (R2 = 0.013), RR (R2 = 0.047)								
TV → IR	0.070 (0.022)	0.115 (0.037)	0.042–0.186	0.002					
TV → RR	0.103 (0.016)	0217 (0.035)	0.144–0.282	<0.001					
IR → CPV	0.560 (0.105)	0.248 (0.047)	0.149–0.336	<0.001					
RR → CPV	1.278 (0.193)	0.436 (0.044)	0.351–0.525	<0.001					
TV → IR → CPV	0.039 (0.013)	0.028 (0.011)	0.010–0.053	0.008					
TV → RR → CPV	0.131 (0.023)	0.095 (0.019)	0.061–0.136	<0.001					
TV → CPV	0.314 (0.048)	0.226 (0.033)	0.158–0.286	<0.001					

Note. DV = direct victimization; VV = vicarious victimization; IRs = instrumental reasons; RRs = reactive reasons; CPV = child-to-parent violence; LLCI = lower limit confidence interval; ULCI = upper limit confidence interval.

## Data Availability

The data supporting this research are open access. You can access them by sending an email to the first author of this article.

## Appendix A

**Table A1:** Factor loadings of the violence exposure scale.

Factor	Item	VES^1^ (Last Year)				VES^2^ (Childhood/Before 10 Years of Age)			
		λ	Mean (*SD*)	SK	KT	λ	Mean (*SD*)	SK	KT
Home	1A	0.767	0.829 (1.146)	1.436	0.624	0.810	1.166 (1.586)	0.911	−0.865
	3A	0.839	0.746 (1.321)	1.595	1.060	0.836	0.912 (1.466)	1.301	0.055
	5A	0.811	1.198 (1.605)	0.878	−0.925	0.899	1.162 (1.641)	0.916	−0.934
	2A	0.759	1.162 (1.433)	0.855	−0.729	0.801	1.468 (1.613)	0.516	−1.374
	4A	0.883	1.245 (1.534)	0.754	−1.026	0.878	1.370 (1.597)	0.615	−1.260
	6A	0.859	1.221 (1.502)	0.831	−0.860	0.943	0.975 (1.481)	1.159	−0.288
School	1B	0.637	1.000 (1.190)	0.866	−0.432	0.748	1.000 (1.190)	0.866	−0.431
	3B	0.751	0.992 (1.217)	0.904	−0.381	0.783	0.992 (1.217)	0.904	−0.381
	5B	0.833	1.211 (1.334)	0.670	−0.858	0.853	1.211 (1.334)	0.670	−0.858
	2B	0.652	0.461 (0.894)	2.048	3.651	0.722	0.411 (0.810)	2.118	4.075
	4B	0.786	0.486 (0.911)	1.924	3.039	0.836	0.583 (0.973)	1.705	2.194
	6B	0.782	0.693 (1.067)	1.482	1.278	0.838	1.144 (1.202)	0.707	−0.601
Street	1C	0.715	1.654 (1.220)	0.051	−1.069	0.721	1.187 (1.276)	0.693	−0.728
	3C	0.831	1.465 (1.292)	0.342	−1.062	0.784	1.027 (1.245)	0.894	−0.404
	5C	0.827	1.931 (1.374)	−0.100	−1.223	0.862	1.231 (1.340)	0.644	−0.902
	2C	0.683	0.260 (0.697)	3.104	9.982	0.730	0.312 (0.787)	2.866	8.129
	4C	0.755	0.449 (0.896)	2.091	3.772	0.817	0.316 (0.771)	2.634	6.581
	6C	0.783	0.725 (1.086)	1.382	0.942	0.831	0.464 (0.932)	2.096	3.695
TV	1D	0.785	2.113 (1.353)	−0.327	−1.113	0.780	1.494 (1.413)	0.324	−1.306
	3D	0.813	1.720 (1.720)	0.059	−1.341	0.837	1.236 (1.357)	0.614	−1.023
	5D	0.831	1.877 (1.421)	−0.052	−1.333	0.828	1.278 (1.433)	0.606	−1.109

Nota. λ = factor loading; SK = skewness; KT = kurtosis.

**Table A2:** Psychometric properties of the VES, CPV-Q, and the reasons subscale for the CPV.

	*x^2^/df*	CFI	TLI	RMSEA	SRMR	ECVI	PNFI	α	ω
VES^1^	581.141/150	0.991	0.987	0.054 (0.050–0.059)	0.049	1.067	0.705	0.89	0.92
VES^2^	584.108/150	0.994	0.991	0.054 (0.050–0.059)	0.046	1.235	0.708	0.92	0.94
CPV-Q^1^	156.198/71	0.989	0.985	0.039 (0.031–0.047)	0.064	0.521	0.734	0.85	0.88
CPV-Q^2^	170.200/91	0.981	0.976	0.044 (0.036–0.052)	0.071	0.530	0.756	0.83	0.86
REA^1^	91.547/91	0.981	0.976	0.070 (0.056–0.084)	0.070	0.320	0.663	0.82	0.89
REA^2^	122.284/19	0.969	0.955	0.083 (0.069–0.097)	0.090	0.306	0.654	0.80	0.88

Note. VES^1^ = Violence Exposure Scale in the last year scale; VES^2^ = Violence Exposure Scale in childhood; CPV-Q^1^ = child-to-parent questionnaire father; CPV-Q^2^ = child-to-parent questionnaire mother; REA^1^ = subscale of reasons for CPV of the father CPV; REA^2^ = subscale of reasons for CPV of the mother.

**Table A3:** Reliability, convergent, and discriminant validity of the VES.

	α_o_	G.6	ω_o_	CR	AVE			HTMT				
						DV Home	VV Home	DV School	VV School	DV Street	VV Street	TV
VES^1^												
DV home	0.85	0.79	0.85	0.791	0.650	1.000						
VV home	0.87	0.83	0.88	0.804	0.698	0.768	1.000					
DV school	0.79	0.71	0.79	0.721	0.554	0.499	0.374	1.000				
VV school	0.78	0.71	0.79	0.755	0.551	0.440	0.367	0.737	1.000			
DV street	0.83	0.77	0.83	0.757	0.632	0.386	0.350	0.877	0.410	1.000		
VV street	0.78	0.71	0.79	0.752	0.555	0.432	0.314	0.591	0.990	0.551	1.000	
TV	0.85	0.79	0.85	0.814	0.656	0.365	0.225	0.500	0.770	0.375	0.833	1.000
VES^2^												
DV home	0.88	0.83	0.88	0.829	0.721	1.000						
VV home	0.91	0.87	0.91	0.844	0.767	0.821	1.000					
DV school	0.84	0.77	0.84	0.765	0.633	0.625	0.447	1.000				
VV school	0.84	0.78	0.84	0.799	0.641	0.538	0.492	0.741	1.000			
DV street	0.83	0.77	0.83	0.733	0.625	0.521	0.527	0.860	0.510	1.000		
VV street	0.83	0.77	0.84	0.793	0.631	0.565	0.432	0.665	0.999	0.555	1.000	
TV	0.85	0.80	0.86	0.812	0.665	0.551	0.356	0.603	0.860	0.458	0.912	1.000

Nota. VES^1^ = violence exposure in the last year scale; VES^2^ = violence exposure in childhood scale; DV = direct victimization; VV = vicarious victimization; α_o_ = Cronbach’s alpha ordinal; G.6 = reliability coefficient G6; ω_o_ = McDonald’s omega ordinal; CR: reliability coefficient; AVE = Average Variance Extracted; HTMT = Heterotrait–Monotrait Ratio.

**Table A4:** Invariance of the Violence Exposure Scale by sex and age of the children.

		CFI	TLI	RMSEA	SRMR	ΔCFI	ΔRMSEA	ΔSRMR
VES last year sex								
M_C_	227.454/160	0.993	0.990	0.029 (0.020–0.038)	0.044			
M_C_ ↔ M_M_	256.046/170	0.991	0.989	0.032 (0.024–0.040)	0.047	0.002	0.003	0.003
M_M_ ↔ M_E_	263.573/180	0.991	0.980	0.031 (0.031–0.039)	0.047	0.000	0.001	0.000
M_E_ ↔ M_S_	274.394/195	0.991	0.991	0.029 (0.020–0.037)	0.049	0.000	0.002	0.002
VES last year age								
M_C_	216.053/160	0.994	0.992	0.027 (0.017–0.035)	0.043			
M_C_ ↔ M_M_	232.373/170	0.993	0.992	0.027 (0.018–0.036)	0.044	0.001	0.000	0.001
M_M_ ↔ M_E_	251.063/180	0.992	0.991	0.028 (0.019–0.036)	0.046	0.001	0.001	0.002
M_E_ ↔ M_S_	261.205/195	0.993	0.992	0.027 (0.017–0.034)	0.048	0.001	0.001	0.012
VES childhood sex								
M_C_	185.994/160	0.998	0.997	0.018 (0.000–0.029)	0.041			
M_C_ ↔ M_M_	204.612/170	0.997	0.997	0.020 (0.006–0.030)	0.043	0.001	0.002	0.002
M_M_ ↔ M_E_	208.295/180	0.998	0.997	0.018 (0.000–0.028)	0.043	0.001	0.002	0.000
M_E_ ↔ M_S_	217.607/195	0.998	0.998	0.015 (0.000–0.026)	0.045	0.000	0.003	0.002
VES childhood age								
M_C_	181.543/160	0.998	0.998	0.017 (0.000–0.027)	0.040			
M_C_ ↔ M_M_	195.557/170	0.998	0.997	0.018 (0.000–0.028)	0.042	0.000	0.001	0.002
M_M_ ↔ M_E_	202.800/180	0.998	0.998	0.016 (0.000–0.027)	0.043	0.000	0.002	0.001
M_E_ ↔ M_S_	217.230/195	0.998	0.998	0.015 (0.000–0.026)	0.044	0.000	0.001	0.001

Nota. M_C_ = configural invariance; M_M_ = metric invariance; M_E_ = strict invariance; M_S_ = scalar invariance.
